# Supplementary material for: Macrophages Use Distinct Actin Regulators to Switch Engulfment Strategies and Ensure Phagocytic Plasticity In Vivo
Source: Cell Rep. 2020 May 26;31(8):107692. doi: 10.1016/j.celrep.2020.107692 (PMC7262594; doi:10.1016/j.celrep.2020.107692)
Supplement: Document S1. Figure S1 [file mmc1.pdf]

Cell Reports, Volume *31*

Supplemental Information

Macrophages Use Distinct Actin Regulators to Switch Engulfment Strategies and Ensure Phagocytic Plasticity *In Vivo*

Andrew J. Davidson and Will Wood

Supplementary Figure 1

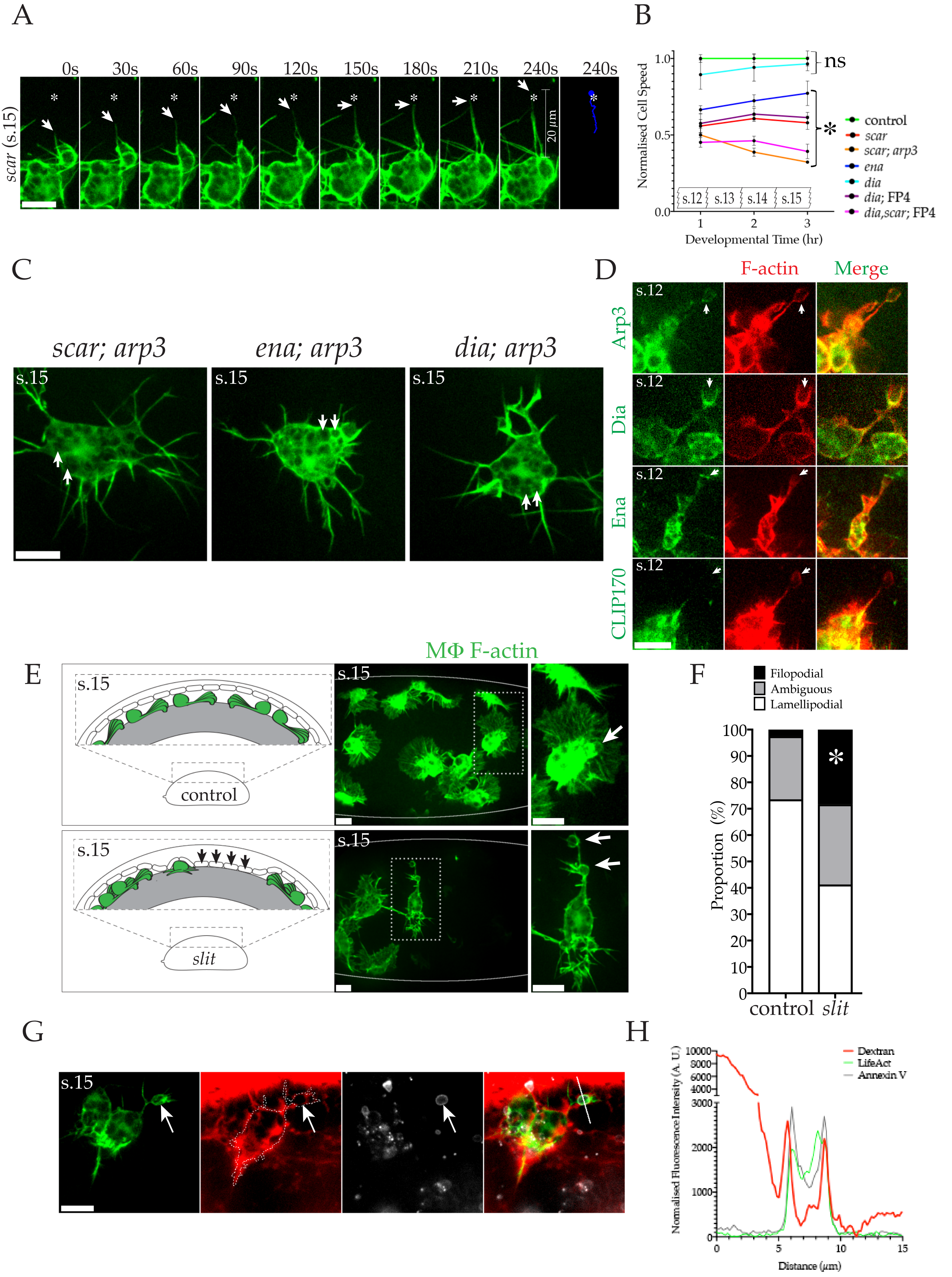

Supplementary Figure Legends

Supplementary Figure 1. Macrophage phagocytosis is very robust and highly plastic

A) Related to Figure 2, phagocytic filopods (Arrows) of *scar* mutant macrophages (LifeAct-GFP, GREEN) are highly directional towards UV-induced apoptotic corpses (*). Final panel shows tracked path (BLUE) of filopod tip across previous panels. Line =peak filopod length (20 μm). Embryonic stage 15. Time =sec.

B) Related to Figure 2, mean cell speed for each hour of development from embryonic stage 12 for indicated genotypes (normalised to control mean at each timepoint). All except *dia* mutants (indicated by 'ns') are significantly slower than the control at all timepoints (indicated by asterisk, ANOVA, $p < 0.05$, 3 embryos/genotype). Error bars =SEM.

C) Related to Figure 3, LifeAct-GFP (GREEN) expressing *scar*; *arp3*, *ena*; *arp3* and *dia*; *arp3* double mutant macrophages. Arrows highlight fluorescent negative vacuoles, indicative of corpse uptake. All embryonic stage 15.

D) Related to Figure 4, localisation of GFP-tagged (GREEN) Arp3 (subunit of Arp2/3 complex), Dia, Ena and CLIP170 (microtubules) with LifeAct-mCherry (F-actin, RED) during Filopodial phagocytosis. Arrows highlight phagocytic filopods. All embryonic stage 12.

E) Related to Figure 4, schematic showing distribution of dispersed macrophages (GREEN) at embryonic stage 15 on ventral side of either control (TOP) or *slit* mutant (BOTTOM) embryos. In *slit* mutants, the extracellular space between the CNS (GREY) and the overlying epithelium fails to open (BLACK Arrows), severely restricting the

dispersal of the macrophages. MIDDLE and RIGHT: Live imaging of phagocytosing control or *slit* mutant macrophages (LifeAct-GFP (F-actin), GREEN). Solid lines mark outline of embryo. Dashed boxes magnified in subsequent panels. Arrows highlight Lamellipodial (control) or Filopodial (*slit*) phagocytic events.

F) Related to Figure 4, percentage of phagocytic events classified as 'Lamellipodial', 'Filopodial' or 'Ambiguous'. Filopodial phagocytosis is significantly more common in *slit* mutant macrophages compared to controls (28.31 ±2.18% vs. 2.50 ±2.50%, mean ±SEM, 5 stage 15 embryos/genotype). Asterisk indicates statistical significance (ANOVA, $p < 0.05$).

G) Related to Figure 4, injection of Dextran (RED) and Annexin V (WHITE) into interstitial space where macrophages reside (MΦ, LifeAct-GFP (F-actin), GREEN) reveals that *slit* mutant macrophages utilising Filopodial phagocytosis (Arrow) are spatially constricted. Dashed line denotes cell outline. Embryonic stage 15.

H) Related to Figure 4, normalised (Background subtracted) fluorescence intensity plots across Filopodial phagocytosis (white line in Fig. S1G) Colour-coded as in (S1G), A. U. =Arbitrary Units.

All scale bars =10 μm, s.12-15 denotes embryonic stage.
