# Supplementary material for: Macrophages Use Distinct Actin Regulators to Switch Engulfment Strategies and Ensure Phagocytic Plasticity In Vivo
Source: Cell Rep. 2020 May 26;31(8):107692. doi: 10.1016/j.celrep.2020.107692 (PMC7262594; doi:10.1016/j.celrep.2020.107692)
Supplement: Document S2. Article plus Supplemental Information [file mmc10.pdf]

Cell Reports

Report

Macrophages Use Distinct Actin Regulators to Switch Engulfment Strategies and Ensure Phagocytic Plasticity *In Vivo*

Graphical Abstract

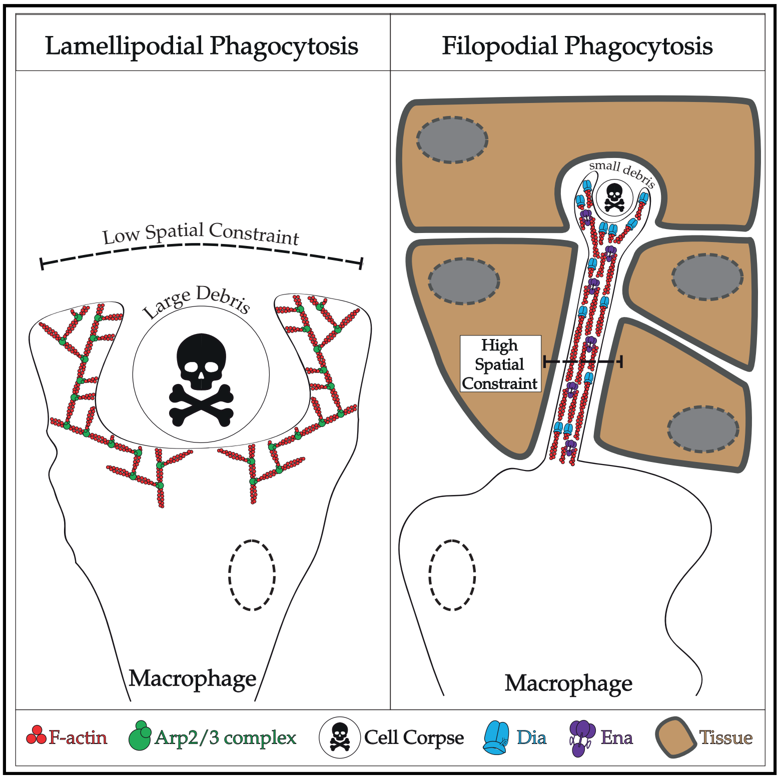

Highlights

- Macrophages use two distinct modes of engulfment: lamellipodial and filopodial phagocytosis
- Arp2/3-complex-dependent lamellipodial phagocytosis involves envelopment via a lamellipod
- Filopodial phagocytosis involves phagocytic filopods extended by Dia and/or Ena
- Macrophages switch to filopodial phagocytosis to overcome spatial constraint *in vivo*

Authors

Andrew J. Davidson, Will Wood

Correspondence

w.wood@ed.ac.uk

In Brief

Through the use of *Drosophila* genetics and the excellent *in vivo* imaging possible in the fly embryo, Davidson and Wood demonstrate that macrophages switch from engulfing debris via sheet-like lamellipods (lamellipodial phagocytosis) to extending phagocytic filopods (filopodial phagocytosis) when spatially confined, thus maintaining their critical clearance function *in vivo*.

Davidson & Wood, 2020, Cell Reports *31*, 107692
May 26, 2020 © 2020 The Authors.
https://doi.org/10.1016/j.celrep.2020.107692

Cell Reports

CellPress
OPEN ACCESS

Report

Macrophages Use Distinct Actin Regulators to Switch Engulfment Strategies and Ensure Phagocytic Plasticity *In Vivo*

Andrew J. Davidson[1] and Will Wood[1,2,*]
[1]Centre for Inflammation Research, University of Edinburgh, Queens Medical Research Institute, 47 Little France Crescent, Edinburgh EH16 4TJ, UK
[2]Lead Contact
*Correspondence: w.wood@ed.ac.uk
https://doi.org/10.1016/j.celrep.2020.107692

SUMMARY

Macrophages must not only be responsive to an array of different stimuli, such as infection and cellular damage, but also perform phagocytosis within the diverse and complex tissue environments found *in vivo*. This requires a high degree of morphological and therefore cytoskeletal plasticity. Here, we use the exceptional genetics and *in vivo* imaging of *Drosophila* embryos to study macrophage phagocytic versatility during apoptotic corpse clearance. We find that macrophage phagocytosis is highly robust, arising from their possession of two distinct modes of engulfment that utilize exclusive suites of actin-regulatory proteins. "Lamellipodial phagocytosis" is Arp2/3-complex-dependent and allows cells to migrate toward and envelop apoptotic corpses. Alternatively, Diaphanous and Ena drive filopodial phagocytosis to reach out and draw in debris. Macrophages switch to "filopodial phagocytosis" to overcome spatial constraint, providing the robust plasticity necessary to ensure that whatever obstacle they encounter *in vivo*, they fulfil their critical clearance function.

INTRODUCTION

Macrophages are highly motile and phagocytic cells that are actively recruited to clear infections and debris arising from development and tissue homeostasis or damage. These professional phagocytes dynamically alter their actin cytoskeleton to drive both their migration toward and engulfment of material. These cytoskeletal rearrangements are coordinated by highly conserved actin regulators. Foremost among these is the Arp2/3 complex, which generates branched actin meshes vital for extending large protrusions such as lamellipods (Mullins et al., 1998; Svitkina and Borisy, 1999). As such, the Arp2/3 complex has been considered vital to both cell motility and phagocytosis (May et al., 2000; Suraneni et al., 2012). However, surprisingly, Arp2/3-deficient murine macrophages have been shown to be both motile and phagocytic (Rotty et al., 2017). Here, we use the powerful genetics of *Drosophila melanogaster* and *in vivo* imaging to investigate Arp2/3-independent modes of macrophage phagocytosis. We demonstrate that macrophage phagocytosis is remarkably robust, not requiring any one or even any combination of the principal actin nucleators (Arp2/3 complex, Ena/VASP, or Diaphanous [Dia] class formins). We delineate two modes of phagocytosis utilized by macrophages, which we term "lamellipodial phagocytosis" and "filopodial phagocytosis." During the latter, macrophages extend finger-like, Arp2/3-independent filopods to draw material back into the cell. We demonstrate that *in vivo*, macrophages resort to filopodial phagocytosis to overcome spatial constriction and reduced mobility so as to maintain their critical clearance function under all circumstances.

RESULTS AND DISCUSSION

Macrophage Phagocytosis Is Extremely Robust

Drosophila macrophages undergo a highly stereotypical migration during embryogenesis (stages 12–15) along the ventral midline of the embryo, where they clear developmental apoptosis by phagocytosis (Tepass et al., 1994; Wood et al., 2006). By the time they are fully dispersed (stage 15), macrophages have efficiently cleared all ventrally placed apoptotic corpses, and these early phagocytic events are vital to "prime" the fly innate immune system, without which macrophages remain unresponsive to wounds (Weavers et al., 2016). To visualize corpse clearance, we injected fluorescent Annexin V into the interstitial space between the overlying epithelium and the developing CNS through which the macrophages disperse (Figure 1A). *In vivo* imaging revealed macrophages used their actin-rich lamellipods to move toward and envelop Annexin-V-labeled apoptotic corpses (Figure 1A; Video S1). The Arp2/3 complex and the sole fly Ena/VASP (Ena) and Dia class formin homologs are collectively the principal actin nucleators within the fly, and we have recently characterized their roles in macrophage motility (Davidson et al., 2019). In order to determine the contribution of these key actin regulators to macrophage

This is an open access article under the CC BY license (http://creativecommons.org/licenses/by/4.0/).

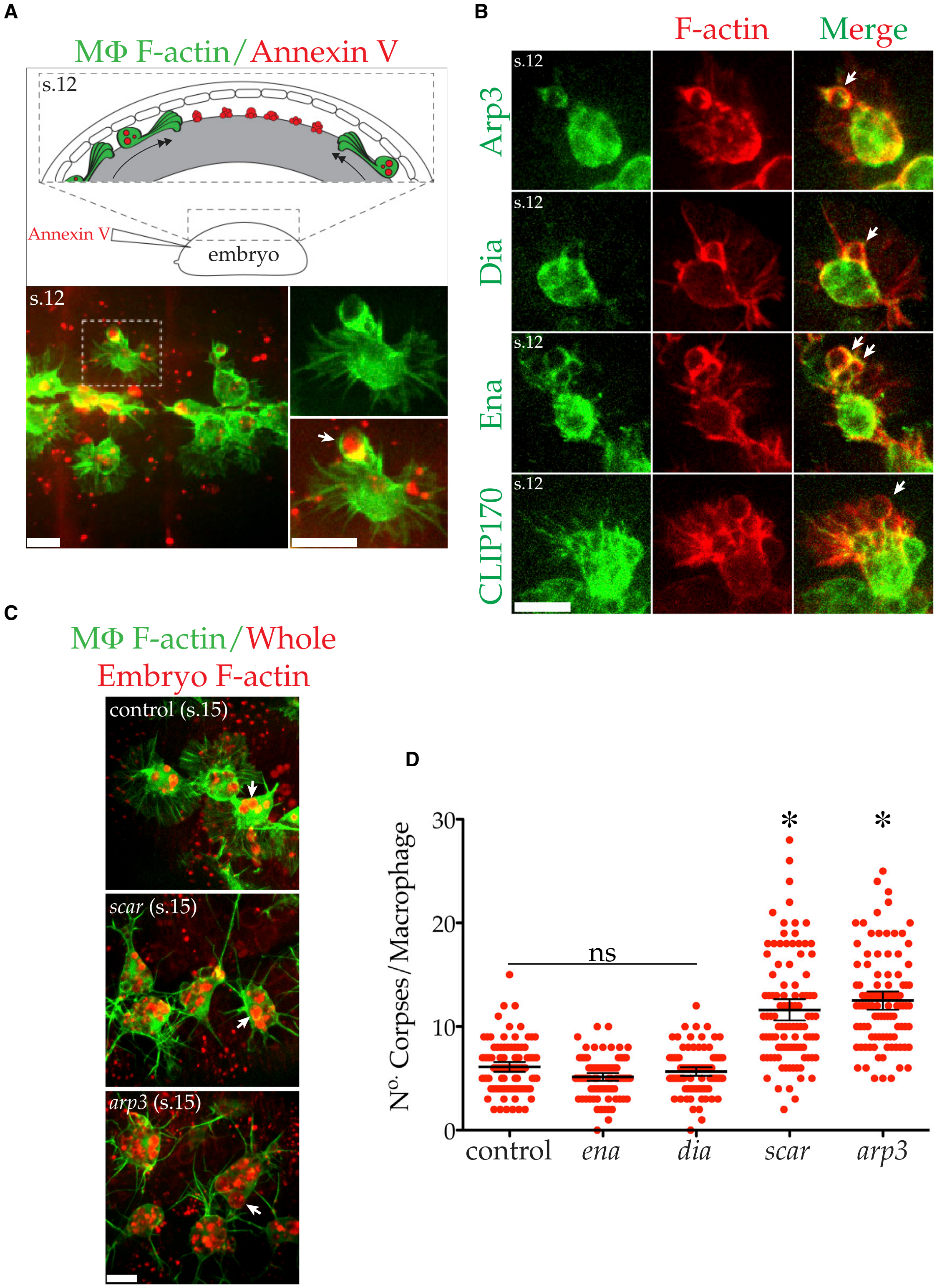

Figure 1. Macrophage Phagocytosis Is Extremely Robust

(A) Top: schematic of the developmental migration of macrophages (MΦ, green) on the ventral side of the *Drosophila* embryo between the CNS (gray) and the overlying epithelium (embryonic stage 12). During their dispersal, macrophages clear corpses, which can be visualized through the injection of fluorescent

(legend continued on next page)

phagocytosis, we explored their localization within macrophages during developmental clearance (stage 12; Figure 1B; Video S2). The Arp2/3 complex was enriched at phagocytic cups, as has been previously observed in mammals (May et al., 2000). However, interestingly, both Ena and Dia also co-localized with F-actin at phagocytic cups within the lamellipods of macrophages as they envelop debris. This was in contrast to microtubules (labeled with GFP-CLIP170), which were absent from phagocytic cups.

Since all three actin nucleators localized to phagocytic cups, we turned to the unrivalled genetics of *Drosophila* to determine which of these actin-regulators were required for engulfment. Clearance of developmentally generated corpses resulted in the concentration of a ubiquitous mCherry probe within macrophages by embryonic stage 15, providing a live reporter of corpse load (Figure 1C). Loss of *ena* or *dia* had no significant effect on corpse load compared to controls (Figure 1D). We have previously shown that *scar* (an activator of the Arp2/3 complex, also known as WAVE) mutant macrophages lack lamellipods and have an elevated corpse load due to a corpse processing defect (Evans et al., 2013). This finding implies that *scar* mutants are still phagocytic despite their lack of lamellipods. We found that both *scar* and *arp3* (an essential Arp2/3 complex subunit) mutant macrophages had significantly increased corpse number when compared to controls (Figure 1D). Therefore, like their murine counterparts, *Drosophila* macrophages are capable of Arp2/3-complex-independent engulfment. Moreover, macrophage phagocytosis is extremely robust and does not require Dia class formins or Ena/VASP.

Arp2/3-Complex-Deficient Macrophages Resort to Filopodial Phagocytosis

Surprisingly, none of the principal actin nucleators were required for macrophage clearance of *in vivo* apoptotic debris. We next sought to challenge the phagocytic ability of these mutants by presenting them with larger corpses to engulf by killing individual ventral epithelial cells with UV irradiation. When combined with fluorescent Annexin V injection, we could visualize the death of these cells and their subsequent clearance *in vivo* (Figure 2A). Individually UV-irradiated epithelial cells are labeled with Annexin V within 5 min, coinciding with the formation of a contractile actin ring within the surrounding epithelial cells, which acts to extrude the dying cell from the epithelial monolayer (Figure 2A). This approach allowed us to precisely control where and when we introduced a cell corpse in the embryo and image the subsequent macrophage recruitment and engulfment. Furthermore, when executed at embryonic stage 15, this UV-induced corpse represents the sole unengulfed debris available to the macrophages.

Control macrophages are recruited to UV-induced corpses within minutes of irradiation and envelop the extruded cell in one or two large phagocytic cups (Figure 2B; Video S3). These phagocytic cups were extremely large, often nearly as large as the macrophage cell body itself (~10 μm in diameter). Loss of *ena* or *dia* had no effect on the ability of macrophages to migrate toward or generate large phagocytic cups (Figure 2B). However, *scar* or *arp3* mutant macrophages responded to UV-induced corpses in an entirely different way. Due to the loss of their lamellipods, *scar* or *arp3* macrophages were impaired in their ability to migrate toward the dead cell. These macrophages instead extended a grasping filopod that drew small pieces of material down the shaft of the filopod, into the cell body (Figures 2B and 2C; Video S4). These phagocytic filopods could reach over twice the length of the cell body (~20 μm) and were extended toward corpses with a high degree of accuracy (Figure S1A). Thus, *scar* or *arp3* macrophages are able to maintain phagocytic ability by using filopods to engage with the corpse instead of lamellipods. This strategy appears to limit phagocytic cup size and therefore restrict the size of particle that can be engulfed. To quantify this, we measured the peak area of every phagocytic cup formed by the different genotypes of macrophages in response to UV-induced cell death (Figure 2D). The majority of all phagocytic cups (including the controls) were no bigger than 25 μm^2 in area at their peak. However, control and *dia* and *ena* mutant macrophages were all also capable of forming extremely large phagocytic cups (>75 μm^2). This reflected a pattern whereby these macrophages combined many smaller phagocytic events with a climactic envelopment of the corpse. However, *scar* and *arp3* macrophages were incapable of forming cups >75 μm^2 in area and were reduced to "nibbling" at the dead cell over a period of hours, likely contributing to their increased corpse load.

Phagocytic filopods such as those observed in *scar* and *arp3* macrophages were also sometimes seen in wild-type macrophages, particularly during dispersal at early stages of development. To further explore the relationship between motility and mode of engulfment, we followed macrophages over the full course of their developmental dispersal (embryonic stages 12–15). Unsurprisingly, loss of the various actin nucleators generally reduced the speed of macrophage dispersal (Figures 2E and S1B). Compared to controls, the dispersal rate of *scar* and double *scar* ; *arp3* mutant macrophages were significantly reduced at all developmental time points (Figure 2E). However, while engulfment through the use of filopods in controls diminished over developmental time, *scar* and double *scar* ; *arp3* mutant

Annexin V (red). Bottom: *in vivo* imaging of dispersing macrophages (stage 12) expressing LifeAct-GFP (F-actin, green) engulfing Annexin-V-labeled debris (red). The dashed box is magnified in subsequent panels. Arrow highlights engulfed corpse.

(B) GFP-tagged Arp3 (subunit of Arp2/3 complex) and Dia and Ena (green) co-localize with LifeAct-mCherry (F-actin, red) at phagocytic cups (in contrast to microtubules [CLIP170]). Arrows highlight actin-rich phagocytic cups (all embryonic stage 12).

(C) Control and *scar* and *arp3* mutant macrophages expressing LifeAct-GFP (F-actin, green) in stage 15 embryos globally expressing mCherry-moesin (F-actin, red). mCherry-labeled corpses (arrows) accumulate in macrophages via engulfment.

(D) Quantification of mCherry-positive corpses/macrophages (control = 6.11 ± 0.24, *dia* = 5.13 ± 0.18, *ena* = 5.66 ± 0.20, *scar* = 11.61 ± 0.52, and *arp3* = 12.51 ± 0.44 [mean corpse/cell ± SEM]; n = 100 cells [>10 stage 15 embryos]/genotype). Error bars represent 95% confidence intervals [CIs], and asterisks indicate statistical significance versus control mean (ANOVA, $p < 0.05$). ns, $p > 0.05$.

All scale bars represent 10 μm. s.12/15 denotes embryonic stage.

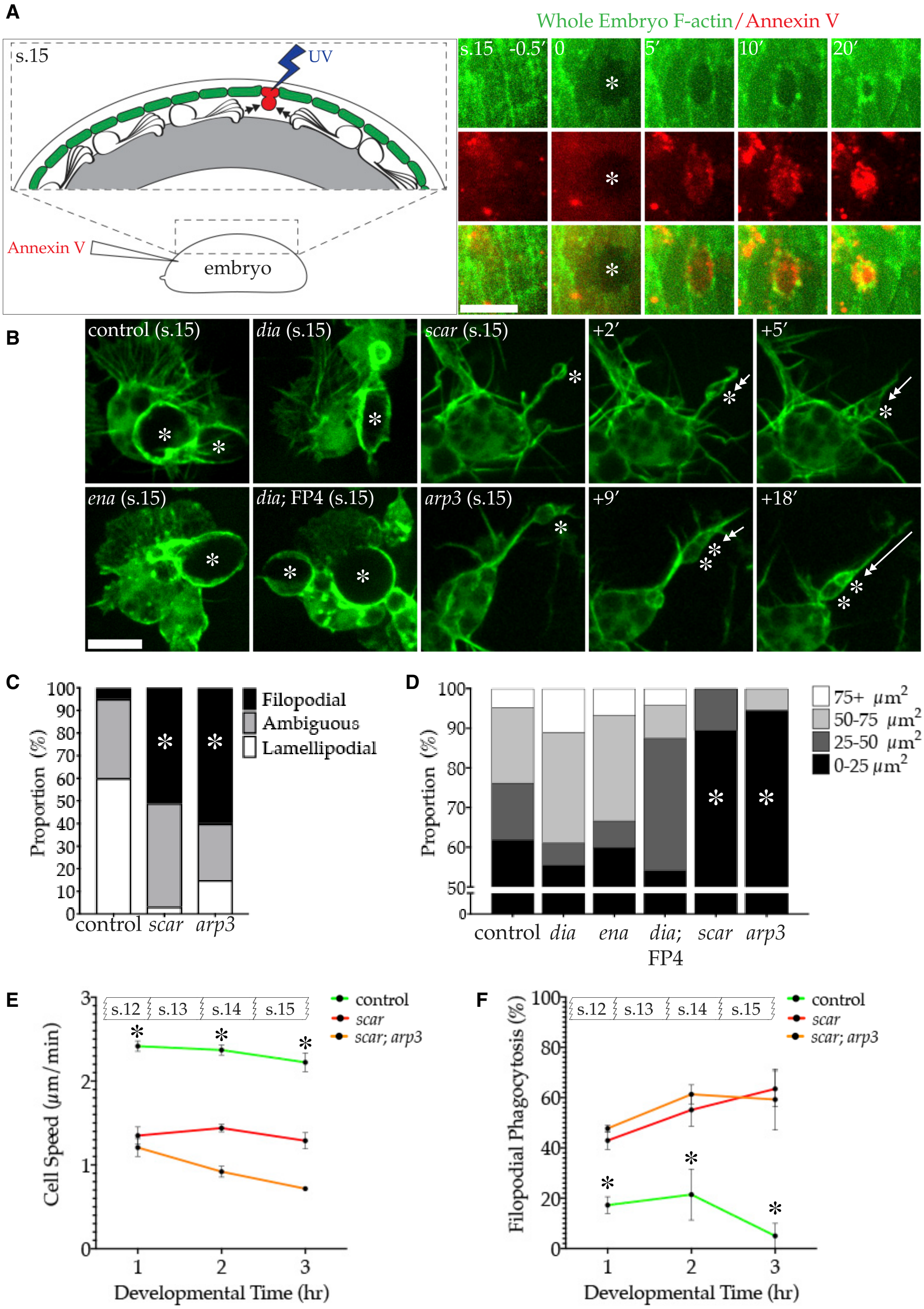

(legend on next page)

macrophages made significantly greater use of such phagocytic protrusions at all time points, correlating with their reduced motility (Figure 2F). It is difficult to separate the pivotal roles played by the lamellipod in cell migration and particle envelopment and they are likely interdependent on one another. Therefore, we cannot definitively conclude that *scar* and *arp3* macrophages utilize phagocytic filopods to overcome their impaired motility. Nevertheless, it appears that in the absence of lamellipods and robust migration, macrophages extend filopods to reach and engulf cell corpses. We designated these two morphologically distinct modes of phagocytosis as Arp2/3-complex-dependent lamellipodial phagocytosis and Arp2/3-complex-independent filopodial phagocytosis.

Dia and Ena Underlie Arp2/3-Complex-Independent Filopodial Phagocytosis

Both Dia and Ena generate linear, unbranched actin filaments found within filopods, and so we tested whether these actin nucleators were responsible for filopodial phagocytosis (Breitsprecher et al., 2008; Pruyne et al., 2002). Both Dia and Ena localized to the phagocytic filopods (both the shaft and the terminal cup) of *scar* mutant macrophages (Figure 3A). This was in contrast to microtubules (GFP-CLIP170), which were absent from the phagocytic cups of *scar* mutants.

Next, we sought to disrupt the filopods (and therefore the phagocytic ability) of *arp3* mutant macrophages by introducing *ena* or *dia* mutations to generate double mutants. However, both *ena*; *arp3* and *dia*; *arp3* double mutants remained highly vacuolated, indicative of proficient engulfment (Figure S1C). In order to quantify corpse load in mutants lacking two or all three of these actin nucleators, we took a different approach so as to overcome the decreasing viability of these flies. We generated double *dia*, *scar* mutants and then inhibited Ena through the macrophage specific expression of the FP4mito construct (Bear et al., 2000). When corpse load was quantified, FP4mito expression (and therefore loss of Ena activity) had no effect on the phagocytic ability of control or *ena* or *dia* mutant macrophages (Figure 3B). This was unsurprising, as all three of these genotypes retain lamellipods and therefore the ability to clear corpses via lamellipodial phagocytosis. Indeed, macrophages lacking both Dia and Ena activity were perfectly capable of enveloping the large corpses generated through UV irradiation (Figures 2B and 2D). In contrast, the elevated corpse load of the *scar* mutant was significantly reduced when combined with the loss of *dia* or with FP4mito expression (Figure 3B). The inhibition of Ena in the double *dia*, *scar* mutant through expression of FP4mito appeared to further reduce macrophage corpse load, although this did not reach statistical significance.

We next quantified mode and rate of engulfment during macrophage dispersal and found that control engulfment steadily declined over developmental time (Figures 3C and 3D). Although the loss of either *dia* or *ena* alone did not alter mode of engulfment, the combined loss of both (*dia*; FP4mito) blocked all developmental filopodial phagocytosis (Figure 3C). This demonstrates that Dia and Ena act together to extend phagocytic filopods in wild-type macrophages during their dispersal. As expected, the overall *dia*; FP4mito engulfment rate was unaffected, since these macrophages retain robust lamellipodial phagocytosis (Figure 3D). In contrast, and consistent with their elevated corpse load, *scar* mutant macrophages have a greatly increased rate of engulfment, peaking during mid-dispersal (Figures 3D). The excessive filopodial phagocytosis and overall elevated engulfment rate of *scar* mutant macrophages was significantly suppressed by the additional loss of both *ena* (via FP4mito expression) and *dia* (Figures 3C and 3D). Remarkably, even the loss of all three of these principal actin nucleators failed to completely block engulfment, although it is possible the residual phagocytosis is supported by the other formins possessed by the fly (Higgs and Peterson, 2005). Nevertheless, given the fact that both Dia and Ena localize to the phagocytic filopods of *scar* mutant macrophages and that the excessive filopodial phagocytosis of *scar* mutant macrophages is suppressed by the disruption of Dia and Ena, we conclude that filopodial phagocytosis is driven by Dia and Ena (Figure 3E).

Wild-Type Macrophages Utilize Filopodial Phagocytosis to Overcome Spatial Restriction

We have shown that Arp2/3-complex-deficient macrophages can resort to filopodial phagocytosis to clear apoptotic debris. We next explored the circumstances under which wild-type

Figure 2. Loss of Arp2/3 Complex Activity Promotes Filopodial Phagocytosis

(A) Left: UV irradiation (blue) of individual epithelial cell (green) induces cell death. The extruded corpse can be visualized through injection of fluorescent Annexin V (red), which is then engulfed by macrophages. Right: series of stills before and after UV irradiation of an individual epithelial cell (*), delineated through global expression of GFP-moesin (F-actin, green). The dying cell is rapidly labeled with Annexin V (red) (embryonic stage 15). Time is in minutes.

(B) Macrophages expressing LifeAct-GFP (green) engulfing UV-induced corpses (*). All but *scar* and *arp3* mutant macrophages are capable of enveloping dying cells with their lamellipods. Instead, *scar* and *arp3* mutant macrophages use filopods to draw small pieces of material (*) back into the cell body (double-headed arrows). FP4mito (FP4) inhibits Ena. All embryonic stage 15. Time is in minutes.

(C) Percentage of phagocytic events classified as lamellipodial, filopodial, or ambiguous in different genotypes in response to UV-induced cell death (control = 5.0 ± 5.0, *scar* = 51 ± 8.72, and *arp3* = 60 ± 18.71 [mean percentage of filopodial phagocytosis ± SEM; five stage 15 embryos/genotype]). Asterisks indicate significantly increased filopodial phagocytosis compared to control (ANOVA, $p < 0.05$).

(D) Peak phagocytic cup size (μm^2) of different genotypes during engulfment of UV-induced apoptotic corpses. y axis represents percentage of phagocytic cups that reached a peak size within a delineated range of areas ($n \geq 15$ phagocytic events [five stage 15 embryos]/genotype). FP4mito (FP4) inhibits Ena. Asterisks indicate significantly reduced peak phagocytic cup size compared to control (ANOVA, $p < 0.05$).

(E) Mean cell speed (μm/min) for each hour of development from embryonic stage 12 for indicated genotypes. Both mutants are significantly slower than the control (*) at all time points (ANOVA, $p < 0.05$, three embryos/genotype). Error bars represent SEM.

(F) Percentage of phagocytic events classified as filopodial for each hour of development from embryonic stage 12 for indicated genotypes. Both mutants exhibit significantly higher frequencies of filopodial phagocytosis than the control (*) at all time points (ANOVA, $p < 0.05$, three embryos/genotype). Error bars represent SEM.

All scale bars represent 10 μm. s.12-15 denotes embryonic stage.

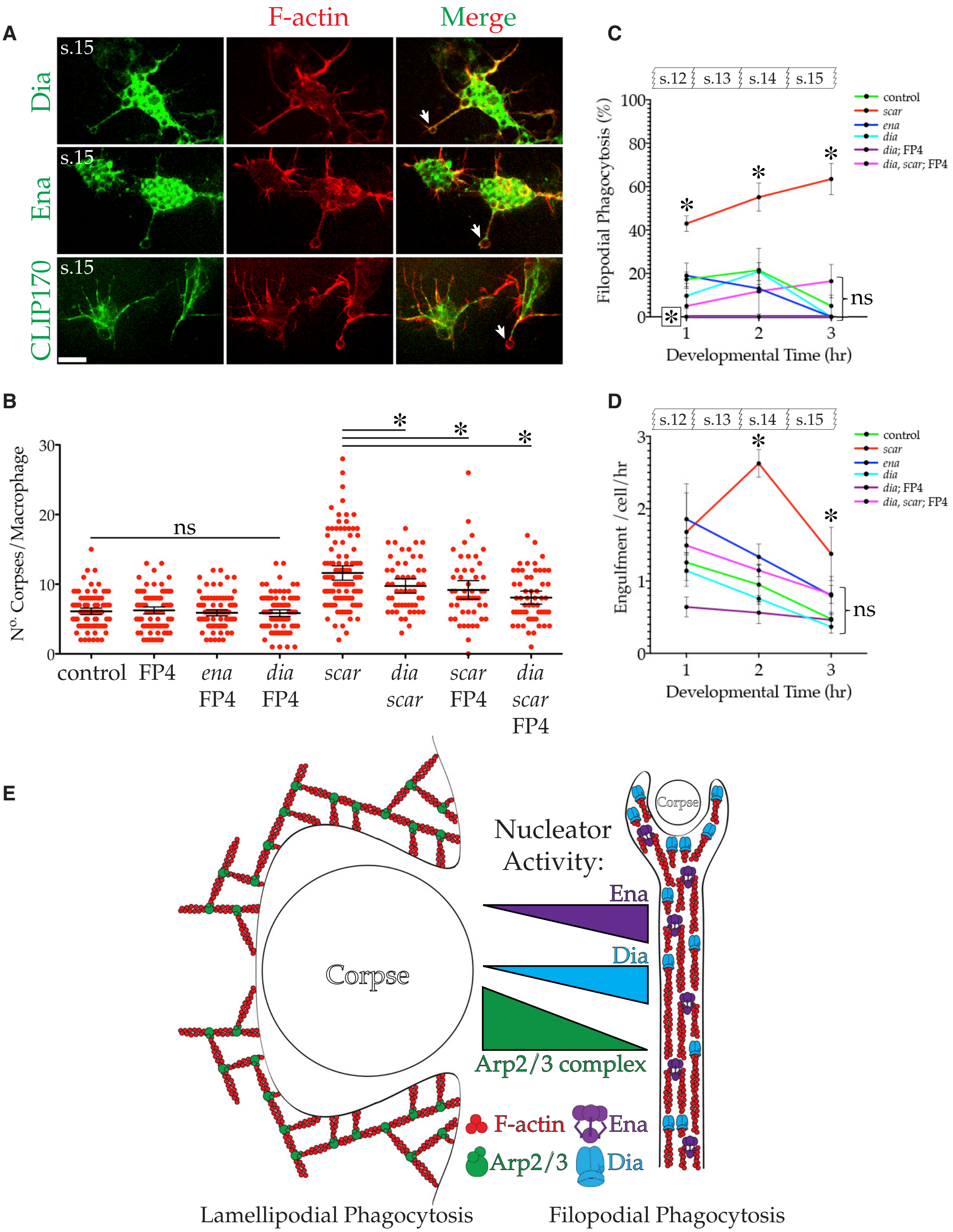

Figure 3. Dia and Ena Underlie Arp2/3-Complex-Independent, Filopodial Phagocytosis

(A) GFP-tagged Dia or Ena (green) co-localize with LifeAct-mCherry (F-actin, red) at filopods and phagocytic cups (arrows) of *scar* mutant macrophages. Microtubules (CLIP170) are present in filopods, but not phagocytic cups. Scale bar represents 10 μm. s.15 denotes embryonic stage 15.

(B) Quantification of mCherry-positive corpses/macrophage (control = 6.11 ± 0.24, FP4mito = 6.24 ± 0.25, *ena*; FP4mito = 5.9 +0.22, *dia*; FP4mito = 5.85 ± 0.24, and *scar* = 11.61 ± 0.52, n = 100 cells [>10 stage 15 embryos]/genotype]; *dia, scar* = 9.75 ± 0.51, *scar*; FP4mito = 9.18 ± 0.66, *dia*, *scar*; and FP4mito = 8.07 ± 0.47, n ≥ 50 cells [≥10 stage 15 embryos]/genotype). Error bars represent 95% CIs, and asterisks indicate statistical significance versus control or *scar* mutant mean (ANOVA, $p < 0.05$). ns, $p > 0.05$. FP4mito (FP4) inhibits Ena activity.

(legend continued on next page)

macrophages utilized either mode of engulfment. First, we imaged macrophages during their dispersal and clearance of apoptotic corpses (stage 12) and confirmed that wild-type macrophages exhibited both lamellipodial and filopodial phagocytosis (Figure 4A; Video S5). We found that wild-type macrophages generally use their lamellipods to migrate toward and engulf cellular debris. However, occasionally, wild-type macrophages would remain stationary and extend filopods to grasp and draw material back into the cell (Figures 4A and 4B). As with lamellipodial phagocytosis, Dia, Ena, and even the Arp2/3 complex localized to the phagocytic protrusion during filopodial phagocytosis (Figures 1B and S1D).

Importantly, we found that these two modes of engulfment were not confined to the clearance of apoptotic debris. Control macrophages were found to internalize bacteria via lamellipodial phagocytosis (Figure 4C). Fully dispersed (stage 15) control macrophages were not observed clearing bacteria through filopodial phagocytosis. However, this is consistent with the infrequent use of phagocytic filopods to engulf apoptotic debris observed with such macrophages (Figures 2C, 2F, and 4B). Instead, in the absence of lamellipodial phagocytosis, bacteria were engulfed through filopodial phagocytosis by *scar* mutant macrophages (Figure 4C). Both modes of engulfment were also evident during the inflammatory response of macrophages to epithelial wounds, where they clear necrotic debris (Figure 4D). We observed some remarkable examples of filopodial phagocytosis occurring at the wound edge, where macrophages would extend extremely long, phagocytic filopods to engulf material further from the wound rather than leave the wound edge (Figure 4D; Video S6). As both lamellipodial phagocytosis and filopodial phagocytosis are deployed during the clearance of apoptotic and necrotic debris as well as bacteria, we propose that both these modes of engulfment can be utilized during the uptake of any type of particle.

Finally, we sought to address why macrophages might adopt one mode of phagocytosis over the other. We have demonstrated that lamellipodial phagocytosis is strongly suppressed in *scar* or *arp3* mutant macrophages, which instead switch to filopodial phagocytosis (Figures 2B, 2C, and 2F). This implies loss of mobility is one reason why macrophages might resort to filopodial phagocytosis. As suggested by our earlier developmental time courses, filopodial phagocytosis was significantly more prevalent in stage 12 macrophages than in stage 15 macrophages (Figure 4B). Live imaging revealed that the macrophages utilizing filopods for engulfment did appear less mobile (Video S5). However, this raised the question as to what was restricting the motility of these specific macrophages as opposed to other cells within the same embryo. The opening of the extracellular space between the epithelium and the CNS through which macrophages migrate precisely coincides with the latter's dispersal and initially retards macrophage migration (Evans et al., 2010a). We therefore hypothesized that dispersing macrophages were adopting filopodial phagocytosis to overcome local spatial constriction, allowing them to engulf apoptotic debris that would otherwise be out of reach.

To test this hypothesis, we initially imaged macrophage engulfment in *slit* mutant embryos, where the separation of the epithelium from the underlying CNS is disrupted and macrophage dispersal is severely impaired (Evans et al., 2010a). Within the anterior region of the embryo, which is not spatially constricted, most *slit* mutant macrophages appeared morphologically normal, retaining both lamellipods, motility, and even lamellipodial phagocytosis (Figure S1E; Video S7). Strikingly however, the most posterior-located macrophage along the CNS (the cell experiencing the greatest spatial constriction) consistently adopted a morphology reminiscent of *scar* or *arp3* mutant macrophages. Furthermore, these *slit* mutant macrophages were consistently extending long, phagocytic filopods (Figure S1E; Video S7). Quantification revealed that filopodial phagocytosis was significantly increased in *slit* mutant macrophages compared to controls (30%; Figure S1F).

To explore this in a wild-type setting, we co-injected fluorescent dextran and Annexin V into the interstitial space, within which the macrophages reside. This allowed us to explore the dynamic relationship between macrophage spatial constraint and the adopted mode of engulfment through three-color live imaging. During developmental dispersal (stage 12), the macrophages are spatially restricted compared to fully dispersed (stage 15) macrophages (Figure 4E). Furthermore, consistent with the diminished rate of engulfment observed with dispersed macrophages, few corpses remain uninternalized to label with Annexin V by this stage (Figures 3D and 4E).

When an apoptotic corpse was in a region of low spatial constraint (visualized by high levels of dextran), it was consistently engulfed through lamellipodial phagocytosis (Figures 4F and 4H; Video S8). In contrast, when an apoptotic corpse was in a region of low dextran (high spatial constraint), it was instead cleared by filopodial phagocytosis (Figures 4G and 4I; Video S8). For example, Annexin-V-labeled corpses were often situated in narrow dextran-filled channels, down which macrophages would extend phagocytic filopods to extract the debris (Figure 4G; Video S8). Spatially constrained *slit* mutant macrophages utilizing filopodial phagocytosis to engulf apoptotic debris were also surrounded by limited dextran (Figures S1G and S1H). Conclusively, when quantified in wild-type embryos, lamellipodial phagocytic events were surrounded by significantly more dextran compared to filopodial phagocytic events (Figure 4J).

We conclude that macrophages have at least two modes of phagocytosis at their disposal. Through their Arp2/3-complex-generated lamellipods, they can migrate toward and envelop a

(C) Percentage of phagocytic events classified as filopodial for each hour of development from embryonic stage 12 for indicated genotypes. *scar* mutant macrophages exhibit significantly higher frequencies of filopodial phagocytosis than the control at all time points (*). ns indicates that none of the other genotypes significantly differ from the control at any time point with the exception of *dia*; FP4mito, which has significantly reduced filopodial phagocytosis at 1 h (boxed asterisk) (ANOVA, $p < 0.05$; three embryos/genotype). Error bars represent SEM.

(D) Engulfment rate per macrophage for each hour of development after embryonic stage 12 for indicated genotypes. *scar* mutants have significantly higher engulfment at indicated (*) time points compared to the control. ns indicates that none of the other genotypes significantly differ from the control at any time point. (ANOVA, $p < 0.05$; three embryos/genotype). Error bars represent SEM.

(E) Schematic illustrating roles of actin regulators in lamellipodial and filopodial phagocytosis.

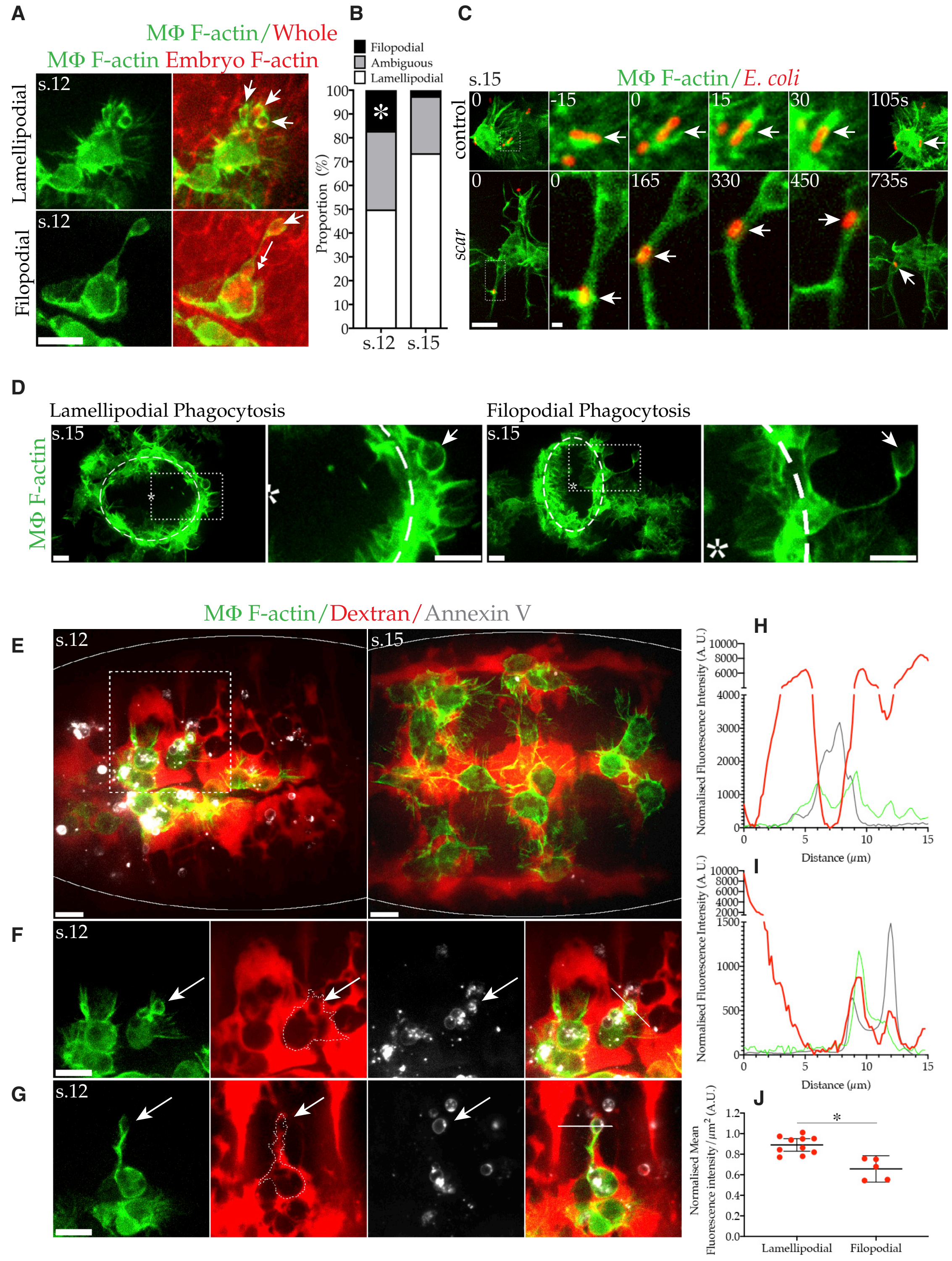

Figure 4. Wild-Type Macrophages Utilize Filopodial Phagocytosis to Overcome Spatial Restriction

(A) Control macrophages expressing LifeAct-GFP (F-actin, green) in stage 12 embryos globally expressing mCherry-moesin (F-actin, red). Control macrophages utilize lamellipodial phagocytosis (top) or filopodial phagocytosis (bottom) to engulf mCherry-positive debris.

(B) Percentage of phagocytic events classified as lamellipodial, filopodial, or ambiguous. Filopodial phagocytosis is significantly more common in dispersing (stage 12) rather than dispersed (stage 15), macrophages (20.41% ± 3.39% versus 2.50% ± 2.50%; mean ± SEM, five stage 15 embryos/genotype). Asterisk indicates statistical significance (ANOVA, $p < 0.05$).

(legend continued on next page)

wide size range of phagocytic targets. Alternatively, when macrophages are spatially constrained, they switch to Arp2/3-complex-independent phagocytic filopods generated by Ena and formins that enable the engulfment of small pieces of otherwise out-of-reach targets.

Conclusion

Like murine macrophage phagocytosis, *Drosophila* macrophage engulfment is remarkably robust and maintained even in the absence of Arp2/3 complex activity. However, through *Drosophila's* powerful genetics, we have been able to penetrate the layers of robustness that are particularly pronounced in mammals but ultimately inherent to the cytoskeleton of all cells. In doing so, we have demonstrated that macrophages utilize Dia and/or Ena to extend phagocytic filopods to overcome the loss of the Arp2/3 complex and the associated loss of lamellipods and motility. Notably, filopods do not appear to play an obvious role in Arp2/3-complex-deficient murine macrophage phagocytosis; however, these cells retain high motility (Rotty et al., 2017). We propose that the formins and Ena/VASP underlie Arp2/3-complex-independent phagocytic cup formation in the macrophages of both flies and mammals. Although lamellipodial phagocytosis appears to be most often favored by the highly motile macrophages studied here, we propose many other less-mobile phagocytes utilize filopodial phagocytosis. For example, embedded tissue-resident macrophages extend protrusions toward cellular damage that appear similar to the phagocytic filopods reported here (Uderhardt et al., 2019).

Importantly, wild-type macrophages utilize both modes of phagocytosis *in vivo* to clear apoptotic debris. We can skew macrophages toward filopodial phagocytosis cell autonomously (through the loss of *scar* or *arp3*) or non-cell autonomously (within a *slit* mutant background).

Although neither the Arp2/3 complex nor Dia or Ena is required for engulfment, each is sufficient to drive the process via either lamellipodial or filopodial phagocytosis, respectively. We also found that all three nucleators localized to the phagocytic cups arising from both modes of engulfment. Therefore, there is normally significant overlap in the activities of the principal nucleators during phagocytosis (Figure 3E). This undoubtedly accounts for the morphological variability observed during engulfment and allows macrophages to fine-tune phagocytosis to meet their immediate needs. Ultimately, this would confer the cytoskeletal plasticity required of macrophages in order to maintain phagocytosis under all of the diverse circumstances encountered *in vivo*.

STAR★METHODS

Detailed methods are provided in the online version of this paper and include the following:

- KEY RESOURCES TABLE
- RESOURCE AVAILABILITY
  - Lead Contact
  - Materials Availability
  - Data Code and Availability
- EXPERIMENTAL MODEL AND SUBJECT DETAILS
  - Fly Stocks
  - Embryo Genotypes
- METHOD DETAILS
  - Live Imaging
- QUANTIFICATION AND STATISTICAL ANALYSIS
- ADDITIONAL RESOURCES

SUPPLEMENTAL INFORMATION

Supplemental Information can be found online at https://doi.org/10.1016/j.celrep.2020.107692.

ACKNOWLEDGMENTS

We would like to thank the Bloomington Stock Center (University of Indiana; NIH P40OD018537) for *Drosophila* stocks, as well as FlyBase. We would also like to thank C. Amato for her help with the Graphical Abstract. This work is funded by a Wellcome Trust senior fellowship to W.W. (107940/Z/15/Z) and a Wellcome Trust Sir Henry Wellcome postdoctoral fellowship to A.J.D. (107355/Z/15/Z)

AUTHOR CONTRIBUTIONS

A.J.D. designed and performed all the experiments, and A.J.D. and W.W. wrote the manuscript.

DECLARATION OF INTERESTS

The authors declare no competing interests.

Received: June 5, 2019
Revised: April 16, 2020
Accepted: May 5, 2020
Published: May 26, 2020

(C) Control (top) and *scar* (bottom) stage 15 macrophages expressing LifeAct-GFP (F-actin, green) engulfing *E. coli* (pHrodo, red) through lamellipodial or filopodial phagocytosis (arrows), respectively. Boxed region enlarged in intervening panels. Time is in seconds; scale bars represent 10/1 μm.
(D) Laser ablation (*) of the embryo (stage 15) leads to the inflammatory recruitment of macrophages expressing LifeAct-GFP (F-actin, green) to the wound edge (dashed line), prompting lamellipodial and filopodial phagocytosis of necrotic debris (arrows). Dotted boxes are magnified in in adjacent panels.
(E–G) Injection of dextran (red) and Annexin V (white) into interstitial space where macrophages (LifeAct-GFP [F-actin], green) reside demonstrates relationship between mode of engulfment and spatial constriction. (E) Dispersing macrophages (stage 12) are surrounded by less dextran-filled space compared to dispersed macrophages (stage 15). Note limited Annexin-V-labeled corpses at stage 15 due to their prior clearance. (F) Dashed box in (E). Lamellipodial phagocytosis of corpse (Arrow) by macrophage in a region of high dextran/low spatial constraint (embryonic stage 12). Dashed line denotes the cell outline. (G) Filopodial phagocytosis of a corpse (arrow) by a macrophage in a region of low dextran/high spatial constraint (embryonic stage 12). Dashed line denotes the cell outline.
(H and I) Normalized (background-subtracted) fluorescence intensity plots across (H) lamellipodial phagocytosis (white line in Figure 4F) or (I) filopodial phagocytosis (white line in Figure 4G). Color-coded as in (F) and (G). A.U., arbitrary units.
(J) Significantly less dextran (mean fluorescence intensity per μm^2, normalized to total dextran signal) surrounds filopodial compared to lamellipodial phagocytic events. A.U., arbitrary units. Asterisk indicates statistical significance (t test, $p < 0.05$, six stage 12 embryos).
Unless otherwise indicated, all scale bars represent 10 μm. s.12/15 denotes embryonic stage.

REFERENCES

Bear, J.E., Loureiro, J.J., Libova, I., Fässler, R., Wehland, J., and Gertler, F.B. (2000). Negative regulation of fibroblast motility by Ena/VASP proteins. Cell *101*, 717–728.

Breitsprecher, D., Kiesewetter, A.K., Linkner, J., Urbanke, C., Resch, G.P., Small, J.V., and Faix, J. (2008). Clustering of VASP actively drives processive, WH2 domain-mediated actin filament elongation. EMBO J. *27*, 2943–2954.

Castrillon, D.H., and Wasserman, S.A. (1994). Diaphanous is required for cytokinesis in Drosophila and shares domains of similarity with the products of the limb deformity gene. Development *120*, 3367–3377.

Davidson, A.J., Millard, T.H., Evans, I.R., and Wood, W. (2019). Ena orchestrates remodelling within the actin cytoskeleton to drive robust *Drosophila* macrophage chemotaxis. J. Cell Sci. *132*, 132.

Evans, I.R., Hu, N., Skaer, H., and Wood, W. (2010a). Interdependence of macrophage migration and ventral nerve cord development in Drosophila embryos. Development *137*, 1625–1633.

Evans, I.R., Zanet, J., Wood, W., and Stramer, B.M. (2010b). Live imaging of Drosophila melanogaster embryonic hemocyte migrations. J. Vis. Exp. (*36*), 1696.

Evans, I.R., Ghai, P.A., Urbančič, V., Tan, K.L., and Wood, W. (2013). SCAR/WAVE-mediated processing of engulfed apoptotic corpses is essential for effective macrophage migration in Drosophila. Cell Death Differ. *20*, 709–720.

Gates, J., Mahaffey, J.P., Rogers, S.L., Emerson, M., Rogers, E.M., Sottile, S.L., Van Vactor, D., Gertler, F.B., and Peifer, M. (2007). Enabled plays key roles in embryonic epithelial morphogenesis in Drosophila. Development *134*, 2027–2039.

Gertler, F.B., Comer, A.R., Juang, J.L., Ahern, S.M., Clark, M.J., Liebl, E.C., and Hoffmann, F.M. (1995). enabled, a dosage-sensitive suppressor of mutations in the Drosophila Abl tyrosine kinase, encodes an Abl substrate with SH3 domain-binding properties. Genes Dev. *9*, 521–533.

Hatan, M., Shinder, V., Israeli, D., Schnorrer, F., and Volk, T. (2011). The Drosophila blood brain barrier is maintained by GPCR-dependent dynamic actin structures. J. Cell Biol. *192*, 307–319.

Higgs, H.N., and Peterson, K.J. (2005). Phylogenetic analysis of the formin homology 2 domain. Mol. Biol. Cell *16*, 1–13.

Homem, C.C., and Peifer, M. (2008). Diaphanous regulates myosin and adherens junctions to control cell contractility and protrusive behavior during morphogenesis. Development *135*, 1005–1018.

Hudson, A.M., and Cooley, L. (2002). A subset of dynamic actin rearrangements in Drosophila requires the Arp2/3 complex. J. Cell Biol. *156*, 677–687.

May, R.C., Caron, E., Hall, A., and Machesky, L.M. (2000). Involvement of the Arp2/3 complex in phagocytosis mediated by FcgammaR or CR3. Nat. Cell Biol. *2*, 246–248.

Mullins, R.D., Heuser, J.A., and Pollard, T.D. (1998). The interaction of Arp2/3 complex with actin: nucleation, high affinity pointed end capping, and formation of branching networks of filaments. Proc. Natl. Acad. Sci. USA *95*, 6181–6186.

Nüsslein-Volhard, C., Wieschaus, E., and Kluding, H. (1984). Mutations affecting the pattern of the larval cuticle inDrosophila melanogaster : I. Zygotic loci on the second chromosome. Wilehm Roux Arch. Dev. Biol. *193*, 267–282.

Pruyne, D., Evangelista, M., Yang, C., Bi, E., Zigmond, S., Bretscher, A., and Boone, C. (2002). Role of formins in actin assembly: nucleation and barbed-end association. Science *297*, 612–615.

Rotty, J.D., Brighton, H.E., Craig, S.L., Asokan, S.B., Cheng, N., Ting, J.P., and Bear, J.E. (2017). Arp2/3 complex is required for macrophage integrin functions but is dispensable for FcR phagocytosis and in vivo motility. Dev. Cell *42*, 498–513.e6.

Stramer, B., Wood, W., Galko, M.J., Redd, M.J., Jacinto, A., Parkhurst, S.M., and Martin, P. (2005). Live imaging of wound inflammation in Drosophila embryos reveals key roles for small GTPases during in vivo cell migration. J. Cell Biol. *168*, 567–573.

Stramer, B., Moreira, S., Millard, T., Evans, I., Huang, C.Y., Sabet, O., Milner, M., Dunn, G., Martin, P., and Wood, W. (2010). Clasp-mediated microtubule bundling regulates persistent motility and contact repulsion in Drosophila macrophages in vivo. J. Cell Biol. *189*, 681–689.

Suraneni, P., Rubinstein, B., Unruh, J.R., Durnin, M., Hanein, D., and Li, R. (2012). The Arp2/3 complex is required for lamellipodia extension and directional fibroblast cell migration. J. Cell Biol. *197*, 239–251.

Svitkina, T.M., and Borisy, G.G. (1999). Arp2/3 complex and actin depolymerizing factor/cofilin in dendritic organization and treadmilling of actin filament array in lamellipodia. J. Cell Biol. *145*, 1009–1026.

Tepass, U., Fessler, L.I., Aziz, A., and Hartenstein, V. (1994). Embryonic origin of hemocytes and their relationship to cell death in Drosophila. Development *120*, 1829–1837.

Thurmond, J., Goodman, J.L., Strelets, V.B., Attrill, H., Gramates, L.S., Marygold, S.J., Matthews, B.B., Millburn, G., Antonazzo, G., Trovisco, V., et al.; FlyBase Consortium (2019). FlyBase 2.0: the next generation. Nucleic Acids Res. *47* (*D1*), D759–D765.

Uderhardt, S., Martins, A.J., Tsang, J.S., Lammermann, T., and Germain, R.N. (2019). Resident macrophages cloak tissue microlesions to prevent neutrophil-driven inflammatory damage. Cell *177*, 541–555.e517.

Weavers, H., Evans, I.R., Martin, P., and Wood, W. (2016). Corpse engulfment generates a molecular memory that primes the macrophage inflammatory response. Cell *165*, 1658–1671.

Wood, W., Jacinto, A., Grose, R., Woolner, S., Gale, J., Wilson, C., and Martin, P. (2002). Wound healing recapitulates morphogenesis in Drosophila embryos. Nat. Cell Biol. *4*, 907–912.

Wood, W., Faria, C., and Jacinto, A. (2006). Distinct mechanisms regulate hemocyte chemotaxis during development and wound healing in Drosophila melanogaster. J. Cell Biol. *173*, 405–416.

Zallen, J.A., Cohen, Y., Hudson, A.M., Cooley, L., Wieschaus, E., and Schejter, E.D. (2002). SCAR is a primary regulator of Arp2/3-dependent morphological events in Drosophila. J. Cell Biol. *156*, 689–701.

Zanet, J., Jayo, A., Plaza, S., Millard, T., Parsons, M., and Stramer, B. (2012). Fascin promotes filopodia formation independent of its role in actin bundling. J. Cell Biol. *197*, 477–486.

STAR★METHODS

KEY RESOURCES TABLE

REAGENT or RESOURCE	SOURCE	IDENTIFIER
Bacterial and Virus Strains		
pHrodo Red *E. coli* bioparticles	Thermo Fisher Scientific, Life Technologies	Cat# P35361
Chemicals, Peptides, and Recombinant Proteins		
VOLTALEF oil	VWR	Cat# 24627.188
Annexin V -Alexa Fluor 568	Molecular Probes, Life Technologies	Cat# A13202
Annexin V -Alexa Fluor 647	Molecular Probes, Life Technologies	Cat# A23204
Dextran, Rhodamine B, 70,000 MW, Neutral	Molecular Probes, Life Technologies	Cat# D1841
Experimental Models: Organisms/Strains		
Drosophila melanogaster: singedGAL4	Zanet et al., 2012	N/A
Drosophila melanogaster: croquemortGAL4	Stramer et al., 2005	N/A
Drosophila melanogaster: uas-gfp-ena	Gates et al., 2007	N/A
Drosophila melanogaster: uas-fp4mito-gfp	Gates et al., 2007	RRID:BDSC_25747
Drosophila melanogaster: uas-lifeact-gfp	Hatan et al., 2011	RRID: BDSC_35544
Drosophila melanogaster: uas-dia-gfp	Homem and Peifer, 2008	N/A
Drosophila melanogaster: uas-lifeact-mcherry	Davidson et al., 2019	N/A
Drosophila melanogaster: uas-gfp-clip170	Stramer et al., 2010	N/A
Drosophila melanogaster: uas-arp3-gfp	Bloomington Drosophila stock center	RRID: BDSC_39723
Drosophila melanogaster: sqh-mcherry-moesin	Bloomington Drosophila stock center	RRID: BDSC_35520, _35521
Drosophila melanogaster: arp3[ep3640]	Hudson and Cooley, 2002	RRID:BDSC_17149
Drosophila melanogaster: dia[2]	Castrillon and Wasserman, 1994	N/A
Drosophila melanogaster: ena[gc1]	Gertler et al., 1995	RRID:BDSC_8569
Drosophila melanogaster: scar[37]	Zallen et al., 2002	RRID:BDSC_8754
Drosophila melanogaster: slit[2]	Nüsslein-Volhard et al., 1984	RRID:BDSC_3266
Software and Algorithms		
Volocity	PerkinElmer	http://cellularimaging.perkinelmer.com/downloads/
ImageJ/FIJI	National Institute of Health (NIH)	http://fiji.sc/
Photoshop	Adobe	http://www.adobe.com/uk/products/photoshop.html
Illustrator	Adobe	http://www.adobe.com/uk/products/illustrator.html
Prism	GraphPad	https://www.graphpad.com/scientific-software/prism/
Excel	Microsoft	https://www.microsoft.com/en-gb/
Other		
UltraView spinning disc microscope	Perkin Elmer	https://www.perkinelmer.com/uk
63x NA1.4 Plan-Apochromat oil objective	Leica	http://www.leica-microsystems.com/home/
C9100-14 Camera	Hamamatsu	https://www.hamamatsu.com/eu/en/product/cameras/emccd-cameras/index.html
Photokinesis FRAPPA unit	Perkin Elmer	https://www.perkinelmer.com/uk
Micropoint ablation laser	Andor Technologies	https://andor.oxinst.com/products/photostimulation/micropoint
Femtojet Injectman Rig	Eppendorf	https://www.eppendorf.com/UK-en/
Femtotip II	Eppendorf	https://www.eppendorf.com/UK-en/

RESOURCE AVAILABILITY

Lead Contact

Further information and requests for resources, reagents, and fly lines should be directed to and will be fulfilled by the Lead Contact, Will Wood (w.wood@ed.ac.uk).

Materials Availability

All fly lines generated in this study will be made available on request by the lead contact with reasonable compensation by the requestor for its shipping.

Data Code and Availability

This study did not generate any unique datasets or code.

EXPERIMENTAL MODEL AND SUBJECT DETAILS

Fly Stocks

SingedGAL4 (*sn-gal4* [Zanet et al., 2012]) and *croquemortGAL4* (*crq-gal4* [Stramer et al., 2005]) were used to drive expression of UAS constructs specifically in macrophages. The following UAS constructs were used in this study: *uas-gfp-ena*, *uas-fp4mito-gfp* (Gates et al., 2007), *uas-lifeact-gfp* (Hatan et al., 2011), *uas-dia-gfp* (Homem and Peifer, 2008), *uas-lifeact-mcherry* (Davidson et al., 2019), *uas-gfp-clip170* (Stramer et al., 2010) *uas-arp3*-gfp (BDSC #39723) and *sqh-mcherry-moesin* (BDSC #35520 and # 35521) The amorphic null alleles used as part of this study were: $arp3^{EP3640}$ (Hudson and Cooley, 2002), dia^{2} (Castrillon and Wasserman, 1994), ena^{GC1}(Gertler et al., 1995), $scar^{37}$(Zallen et al., 2002) and $slit^{2}$ (Nüsslein-Volhard et al., 1984). The majority of all fly lines used as part of this work were derived from those ordered through the Bloomington Stock Centre (University of Indiana, USA (NIH P40OD018537). FlyBase (Thurmond et al., 2019) was also used extensively for genetic and molecular information.

Embryo Genotypes

The following table lists the fly strains used in this study in order of first appearance:

; sn-gal4, uas-lifeact-gfp
; sn-gal4, uas-arp3-gfp ; sn-gal4, uas-lifeact-mcherry
; sn-gal4, uas-dia-gfp ; sn-gal4, uas-lifeact-mcherry
; sn-gal4, uas-lifeact-mcherry ; crq-gal4, uas-ena-gfp
; sn-gal4, uas-lifeact-mcherry ; uas-clip170-GFP/ sn-gal4, uas-lifeact-mcherry
; sn-gal4, uas-lifeact-gfp; sqh-mcherry-moesin
; $scar^{37}$, *sn-gal4, uas-lifeact-gfp ; sqh-mcherry-moesin*
; sqh-mcherry-moesin ; $arp3^{EP3640}$, *sn-gal4, uas-lifeact-gfp*
;; ubi-gfp-moesin
; dia^{2}, *sn-gal4, uas-lifeact-gfp ; sqh-mcherry-moesin*
; ena^{GC1}, *sn-gal4, uas-lifeact-gfp ; sqh-mcherry-moesin*
; dia^{2}, *sn-gal4, uas-lifeact-gfp ; crq-gal4, uas-FP4mito-gfp/+*
; dia^{2}, *sn-gal4, uas-lifeact-gfp ; crq-gal4, uas-FP4mito-gfp,sqh-mcherry-moesin*
; $scar^{37}$, *sn-gal4, uas-dia-gfp ; sn-gal4, uas-lifeact-mcherry*
; $scar^{37}$, *sn-gal4, uas-lifeact-mcherry ; crq-gal4, uas-ena-gfp*
; $scar^{37}$, *sn-gal4, uas-lifeact-mcherry ; crq-gal4, uas-ena-gfp*
; $scar^{37}$, *sn-gal4, uas-lifeact-mcherry ; uas-clip170-gfp/+*
; $scar^{37}$; $arp3^{EP3640}$, *sn-gal4, uas-lifeact-gfp*
; ena^{GC1} ; $arp3^{EP3640}$, *sn-gal4, uas-lifeact-gfp*
; dia^{2} ; $arp3^{EP3640}$, *sn-gal4, uas-lifeact-gfp*
; sn-gal4, uas-lifeact-gfp ; crq-gal4, uas-FP4mito-gfp,sqh-mcherry-moesin
; ena^{GC1}, *sn-gal4, uas-lifeact-gfp ; crq-gal4, uas-FP4mito-gfp,sqh-mcherry-moesin*
; dia^{2}, $scar^{37}$, *sn-gal4, uas-lifeact-gfp ; sqh-mcherry-moesin*
; $scar^{37}$, *sn-gal4, uas-lifeact-gfp ; crq-gal4, uas-FP4mito-gfp,sqh-mcherry-moesin/sqh-mcherry-moesin*
; dia^{2}, $scar^{37}$, *sn-gal4, uas-lifeact-gfp ; crq-gal4, uas-FP4mito-gfp/+*
; dia^{2}, $scar^{37}$, *sn-gal4, uas-lifeact-gfp ; crq-gal4, uas-FP4mito-gfp,sqh-mcherry-moesin/sqh-mcherry-moesin*
; $slit^{2}$; *sn-gal4, uas-lifeact-gfp*

METHOD DETAILS

Live Imaging

Embryos laid overnight at 25°C were collected in cell strainers (Falcon), dechorionated with bleach (Jangro), washed repeatedly with water and embryos at developmental stage 12 or 15 were mounted between a glass slide and a supported coverslip in droplets of VOLTALEF oil (VWR) (Evans et al., 2010b). Z stacks (20x 0.5 μm slices) of the ventral macrophages were then acquired using a spinning disc confocal microscope (Perkin Elmer Ultraview) with a plan-apochromat 63x objective with a NA of 1.4 and a Hamamatsu C9100-14 camera. The acquisition software used was Volocity (Perkin Elmer). Epithelial apoptosis was induced by targeted UV-irradiation using the FRAPPA unit (PhotoKinesis™ module) on the UltraVIEW spinning disc system. Global, Gal4-independent expression of an F-actin probe (GMA/mCherry-Moesin) delineated the epithelial boundaries and was used to target individual cells. Cells were irradiated using the 405 nm laser (100%) for 300 cycles of 100 ms bleaches (~40 s total) using the crosshair at the smallest spot size. For the injection of fluorescent dyes/bacteria into embryos, embryos were dechorionated, washed, and mounted as normal before being dehydrated in a sealed box with silica beads for ~15-20 mins. A droplet of VOLTALEF oil (VWR) was added to each embryo before anterior injection with either undiluted Annexin V-Alexa Fluor 568 (Molecular probes, Life Technologies), Rhodamin B dextran (70 kDa, Molecular probes, Life Technologies) diluted 1 in 10 with Annexin V-Alexa Fluor 647 (Molecular probes, Life Technologies) or pHrodo Red *E. coli* Bioparticles (Thermo Fisher Scientific, Life Technologies) diluted with PBS at a concentration of 2 mg/mL. The injection was performed using a FemtoJet injectman rig (Eppendorf) fitted with Femto tips (Eppendorf). A coverslip was sealed on top and imaging commenced. Epithelial wounds were generated using laser ablation (nitrogen-pumped micropoint ablation laser tuned to 435 nm, Andor Technologies) as previously described (Wood et al., 2002).

Although the actin-binding probe, LifeAct, was used in the majority of images throughout this manuscript, similar protrusion dynamics were observed with other cytoskeletal probes (e.g., GFP-Moesin) and with cytosolic GFP.

QUANTIFICATION AND STATISTICAL ANALYSIS

All image analysis was performed in ImageJ (NIH). All the images present in the figures and movies of this manuscript are maximum intensity z-projections. Cell centroids were tracked manually using the ImageJ Manual tracking plugin. For corpse load analysis, macrophages were outlined in the GFP-channel and then mCherry-positive corpses were counted manually within this outline. The unprojected z stack was used to ensure the corpses were within the macrophages. To calculate peak phagocytic cup area, the maximum LifeAct-GFP cup size reached for every engulfment event during clearance of UV-generated corpses was identified within the unprojected z stack (0.5 μm slices) and the area measured using the ImageJ measure tool.

For quantification of mode and rate of engulfment, we defined Lamellipodial phagocytosis as engulfment involving a phagocytic cup surrounded by a sheet-like protrusion (lamellipod). We defined Filopodial Phagocytosis as engulfment involving a phagocytic cup projecting from a finger-like protrusion (filopod) and devoid of all lamellipod. We defined Ambiguous phagocytosis as either sharing elements of both Lamellipodial and Filopodial phagocytosis, elements of neither or when the engulfment was partially obscured form view. Using these definitions, we manually classified phagocytic events as either Lamellipodial, Filopodial or Ambiguous by analyzing timelapses frame-by-frame in ImageJ. Macrophages often engulfed corpses through multiple phagocytic cups formed within several time frames of each other as part of the same protrusion and were counted as a single phagocytic event. For example, the Lamellipodial and Filopodial phagocytosis shown Figure 4A and Video S5 were both counted as one instance of engulfment.

Fluorescence intensity plots were generated in ImageJ by using the line tool to draw a 15 μm long, 10 pixel wide (only central 2 pixels shown in figure so as not to obscure image) line across phagocytic cups and using the plot profile tool. For each channel, the lowest value (the background) was subtracted from the profile. To quantify spatial constriction, a 200 μm^2 circle was drawn around the macrophage, centered on the phagocytic cup. The mean fluorescence intensity of the Rhodamine B dextran within this circle was then calculated using the measure tool in imageJ. This value was normalized to the mean dextran fluorescence within the entire interstitial space.

Unpaired, two-tailed t tests and one-way ANOVA with a Tukey's or Dunnett's multiple comparisons test were used to test statistical significance and generate p values using GraphPad Prism software. Details of the statistical tests conducted for each experiment and their output is reported in the figure legends.

ADDITIONAL RESOURCES

None.

Cell Reports, Volume *31*

Supplemental Information

Macrophages Use Distinct Actin Regulators to Switch Engulfment Strategies and Ensure Phagocytic Plasticity *In Vivo*

Andrew J. Davidson and Will Wood

Supplementary Figure 1

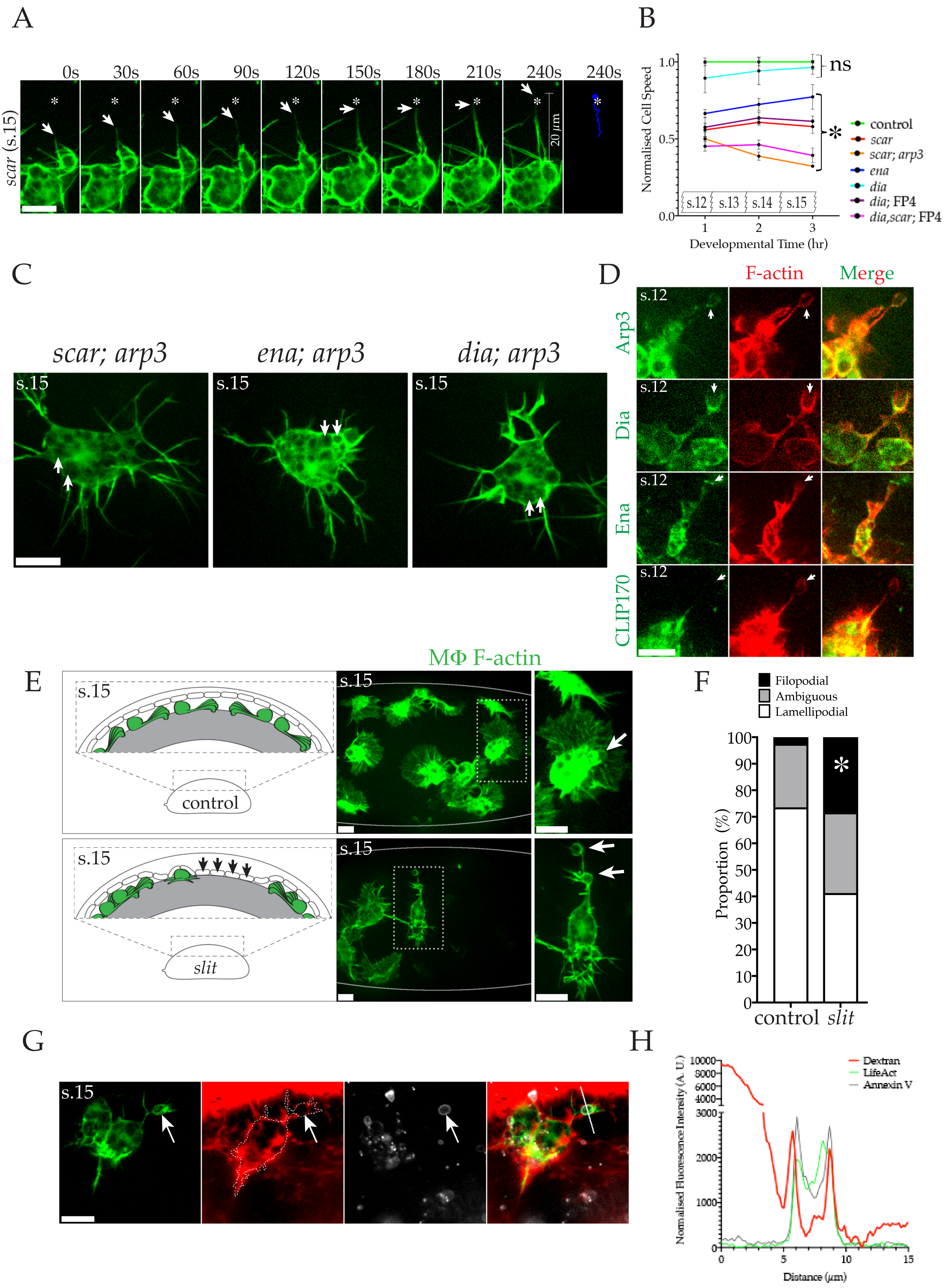

Supplementary Figure Legends

Supplementary Figure 1. Macrophage phagocytosis is very robust and highly plastic

A) Related to Figure 2, phagocytic filopods (Arrows) of *scar* mutant macrophages (LifeAct-GFP, GREEN) are highly directional towards UV-induced apoptotic corpses (*). Final panel shows tracked path (BLUE) of filopod tip across previous panels. Line =peak filopod length (20 μm). Embryonic stage 15. Time =sec.

B) Related to Figure 2, mean cell speed for each hour of development from embryonic stage 12 for indicated genotypes (normalised to control mean at each timepoint). All except *dia* mutants (indicated by 'ns') are significantly slower than the control at all timepoints (indicated by asterisk, ANOVA, $p < 0.05$, 3 embryos/genotype). Error bars =SEM.

C) Related to Figure 3, LifeAct-GFP (GREEN) expressing *scar*; *arp3*, *ena*; *arp3* and *dia*; *arp3* double mutant macrophages. Arrows highlight fluorescent negative vacuoles, indicative of corpse uptake. All embryonic stage 15.

D) Related to Figure 4, localisation of GFP-tagged (GREEN) Arp3 (subunit of Arp2/3 complex), Dia, Ena and CLIP170 (microtubules) with LifeAct-mCherry (F-actin, RED) during Filopodial phagocytosis. Arrows highlight phagocytic filopods. All embryonic stage 12.

E) Related to Figure 4, schematic showing distribution of dispersed macrophages (GREEN) at embryonic stage 15 on ventral side of either control (TOP) or *slit* mutant (BOTTOM) embryos. In *slit* mutants, the extracellular space between the CNS (GREY) and the overlying epithelium fails to open (BLACK Arrows), severely restricting the

dispersal of the macrophages. MIDDLE and RIGHT: Live imaging of phagocytosing control or *slit* mutant macrophages (LifeAct-GFP (F-actin), GREEN). Solid lines mark outline of embryo. Dashed boxes magnified in subsequent panels. Arrows highlight Lamellipodial (control) or Filopodial (*slit*) phagocytic events.

F) Related to Figure 4, percentage of phagocytic events classified as 'Lamellipodial', 'Filopodial' or 'Ambiguous'. Filopodial phagocytosis is significantly more common in *slit* mutant macrophages compared to controls (28.31 ±2.18% vs. 2.50 ±2.50%, mean ±SEM, 5 stage 15 embryos/genotype). Asterisk indicates statistical significance (ANOVA, $p < 0.05$).

G) Related to Figure 4, injection of Dextran (RED) and Annexin V (WHITE) into interstitial space where macrophages reside (MΦ, LifeAct-GFP (F-actin), GREEN) reveals that *slit* mutant macrophages utilising Filopodial phagocytosis (Arrow) are spatially constricted. Dashed line denotes cell outline. Embryonic stage 15.

H) Related to Figure 4, normalised (Background subtracted) fluorescence intensity plots across Filopodial phagocytosis (white line in Fig. S1G) Colour-coded as in (S1G), A. U. =Arbitrary Units.

All scale bars =10 μm, s.12-15 denotes embryonic stage.
